# Automated abdominal organ segmentation algorithms for non-enhanced CT for volumetry and 3D radiomics analysis

**DOI:** 10.1007/s00261-024-04581-5

**Published:** 2024-09-19

**Authors:** Junghoan Park, Ijin Joo, Sun Kyung Jeon, Jong-Min Kim, Sang Joon Park, Soon Ho Yoon

**Affiliations:** 1 Seoul National University, Seoul, Republic of Korea; 2 Seoul National University Hospital, Seoul, Republic of Korea; 3 MEDICAL IP. Co., Ltd, Seoul, Republic of Korea

**Keywords:** Deep Learning, Neural Networks, Computer, Organ Size, Radiomics, Tomography, X-Ray Computed

## Abstract

**Purpose:**

To develop fully-automated abdominal organ segmentation algorithms from non-enhanced abdominal CT and low-dose chest CT and assess their feasibility for automated CT volumetry and 3D radiomics analysis of abdominal solid organs.

**Methods:**

Fully-automated nnU-Net-based models were developed to segment the liver, spleen, and both kidneys in non-enhanced abdominal CT, and the liver and spleen in low-dose chest CT. 105 abdominal CTs and 60 low-dose chest CTs were used for model development, and 55 abdominal CTs and 10 low-dose chest CTs for external testing. The segmentation performance for each organ was assessed using the Dice similarity coefficients, with manual segmentation results serving as the ground truth. Agreements between ground-truth measurements and model estimates of organ volume and 3D radiomics features were assessed using the Bland–Altman analysis and intraclass correlation coefficients (ICC).

**Results:**

The models accurately segmented the liver, spleen, right kidney, and left kidney in abdominal CT and the liver and spleen in low-dose chest CT, showing mean Dice similarity coefficients in the external dataset of 0.968, 0.960, 0.952, and 0.958, respectively, in abdominal CT, and 0.969 and 0.960, respectively, in low-dose chest CT. The model-estimated and ground truth volumes of these organs exhibited mean differences between − 0.7% and 2.2%, with excellent agreements. The automatically extracted mean and median Hounsfield units (ICCs, 0.970–0.999 and 0.994–0.999, respectively), uniformity (ICCs, 0.985–0.998), entropy (ICCs, 0.931–0.993), elongation (ICCs, 0.978–0.992), and flatness (ICCs, 0.973–0.997) showed excellent agreement with ground truth measurements for each organ; however, skewness (ICCs, 0.210–0.831), kurtosis (ICCs, 0.053–0.933), and sphericity (ICCs, 0.368–0.819) displayed relatively low and inconsistent agreement.

**Conclusion:**

Our nnU-Net-based models accurately segmented abdominal solid organs in non-enhanced abdominal and low-dose chest CT, enabling reliable automated measurements of organ volume and specific 3D radiomics features.

**Supplementary Information:**

The online version contains supplementary material available at 10.1007/s00261-024-04581-5.

## Introduction

Three-dimensional (3D) organ segmentation in medical imaging is fundamental in many clinical and research applications because it enables image-based organ analysis. Organ volumetry is a diagnostic or prognostic marker for various abdominal diseases. Previous studies have revealed that preoperative liver volume is an important predictor for postoperative hepatic failure [1, 2]. Spleen volume or liver-to-spleen volume ratio is associated with hepatic fibrosis severity [3, 4] and patient outcomes in liver cirrhosis [4–6]. Kidney volume can predict the glomerular filtration rate in chronic kidney disease [7] and is a prognostic marker in autosomal dominant polycystic kidney disease [8]. Additionally, organ segmentation can provide radiomics features of the segmented organs. Radiomics is a rapidly growing field involving the extraction of quantitative data from medical images [9] and can be used for differential diagnosis, risk stratification, and treatment monitoring. One well-established example of organ radiomics in clinical use is hepatic steatosis evaluation using CT attenuation values of the liver and spleen [10, 11]. Additionally, recent studies have suggested that radiomics features of the liver parenchyma may predict hepatic fibrosis presence and severity [12, 13].

Non-enhanced abdominal CT is a basic imaging modality for abdominal organ segmentation that has been actively studied [14–17]. Non-enhanced images are commonly included in various abdominal CT protocols used in practice, providing advantages such as data availability and future applicability. In addition, non-enhanced abdominal CT may be more reliable than contrast-enhanced CT for assessing radiomics features, as it allows for the analysis of tissue characteristics without the variations introduced by contrast agent dose, concentration, or scan timing. Indeed, CT attenuation values, one of the most widely used radiomics features, have been shown to provide a more accurate diagnosis of hepatic steatosis in non-enhanced CT compared to contrast-enhanced CT [10, 11]. Furthermore, low-dose chest CT, a screening tool for lung cancer, covers a substantial portion of the upper abdomen. Evaluating all abdominal organs is infeasible in low-dose chest CT; however, low-dose chest CT can provide information about the upper abdominal organs. Therefore, segmenting abdominal organs on low-dose chest CT may serve as a substitute for abdominal CT in certain indications. A recent study reported the utility of CT attenuation values of the liver measured on low-dose chest CT for identifying moderate-to-severe hepatic steatosis [18].

Recent advances in deep learning techniques based on convolutional neural networks have shown promising results in fully-automated organ segmentation in CT or MRI images [19]. The nnU-Net algorithm is a cutting-edge deep learning approach that utilizes a cascade of convolutional neural networks to achieve more precise and efficient analyses [20]. This makes it beneficial for large cohort studies where manual segmentation is infeasible. However, the performance of fully-automated organ segmentation techniques primarily focuses on segmentation accuracy [21–23]. In contrast, data on the accuracy of extracted values, such as organ volume and radiomics features of the segmented organs, are relatively scarce. Therefore, evaluating their robustness and reliability is essential because of the evolving applications of automatically extracted imaging parameters.

This study aimed to develop and evaluate fully-automated multi-organ segmentation algorithms from non-enhanced abdominal CT and low-dose chest CT and assess their feasibility for automated CT volumetry and 3D radiomics analysis of abdominal solid organs.

## Materials and methods

The institutional review board of Seoul National University Hospital approved this study, and the requirement for informed consent was waived because of the retrospective nature of the study. ChatGPT was used for English grammar and expression corrections, but it did not affect the overall content of the paper.

### Data sources

Two CT scan datasets were used for model development: 105 abdominal CT scans for the abdominal CT model and 60 low-dose chest CT scans for the low-dose chest CT model. Additionally, external test datasets comprising 55 abdominal CT and 10 low-dose chest CT scans were used to assess the performance of these models. The CT acquisition and reconstruction parameters for non-enhanced abdominal CT and low-dose chest CT used in the development and external test sets are presented in Supplementary tables 1 and 2.

#### Non-enhanced abdominal CT scans

The model development included 105 abdominal CT scans that incorporated non-enhanced phase images obtained from 105 patients. These scans were conducted in the emergency department of our institution between June and November 2020 using a multidetector CT machine (Aquilion ONE, Canon Medical Systems). The inclusion criteria comprised patients aged ≥ 18 years who had not undergone surgical resection or interventional treatment on any of the target organs (the liver, spleen, right kidney, and left kidney) and had no mass-like lesions that altered the normal organ contours. Patients with large or extensive cysts, such as those with autosomal dominant polycystic kidney disease, visible on non-enhanced CT, were excluded. However, organ size was not applied as a separate inclusion or exclusion criterion. The external test dataset consisted of 55 non-enhanced abdominal CT scans acquired from 55 patients at our institution using CT machines different from those used in the development set.

#### Low-dose chest CT scans

Model development included a dataset of 60 low-dose chest CT scans from 60 patients obtained during health check-ups at our institution between January and February 2021. The inclusion criteria for selecting the low-dose chest CT scans matched those used for the abdominal CT dataset, with a focus on the liver and spleen as target organs. While the scan range of low-dose chest CT typically includes a significant portion of the liver and spleen, it does not always capture their lower edges. Therefore, patients with incomplete scans of these organs were included in the study, and only the portions of the liver and spleen within the scan range were evaluated.. The low-dose chest CT scans for the development dataset were obtained using three different CT machines, including Somatom Force (Siemens Healthineers, n = 25), Somatom Definition (Siemens Healthineers, n = 20), and iCT 256 (Philips Healthcare, n = 15). For the external test dataset, 10 low-dose chest CT scans from 10 patients acquired at our institution using a CT machine (Discovery CT750 HD, GE Healthcare) different from those used for the development set.

### Data preparation

All CT data in the development and external test sets were initially processed using a commercially available software program (MEDIP PRO, v2.0.0, MEDICAL IP Co., Ltd., Seoul, Korea) to efficiently obtain the 3D organ label for each target organ in CT images. The target organs included the liver, spleen, right kidney, and left kidney for abdominal CT, whereas only the liver and spleen were targeted for low-dose chest CT. A board-certified radiologist (J.P., with 6 years of experience in body CT interpretation) manually adjusted the preliminary labels slice-by-slice to create the ground truth (GT). Several rules were applied during the process: (1) the hepatic fissures, intrahepatic portion of the inferior vena cava, and large portal veins (such as the main and lobar portal veins) were excluded from the liver mask as much as possible; (2) only the renal cortex and medulla, excluding the renal sinus, were included in the kidney mask; and (3) all focal lesions within the organs were included in the organ mask, considering the limitations of non-enhanced CT in identifying and delineating focal lesions.

### Development of 3D nnU-Net models

**Fig. 1:**
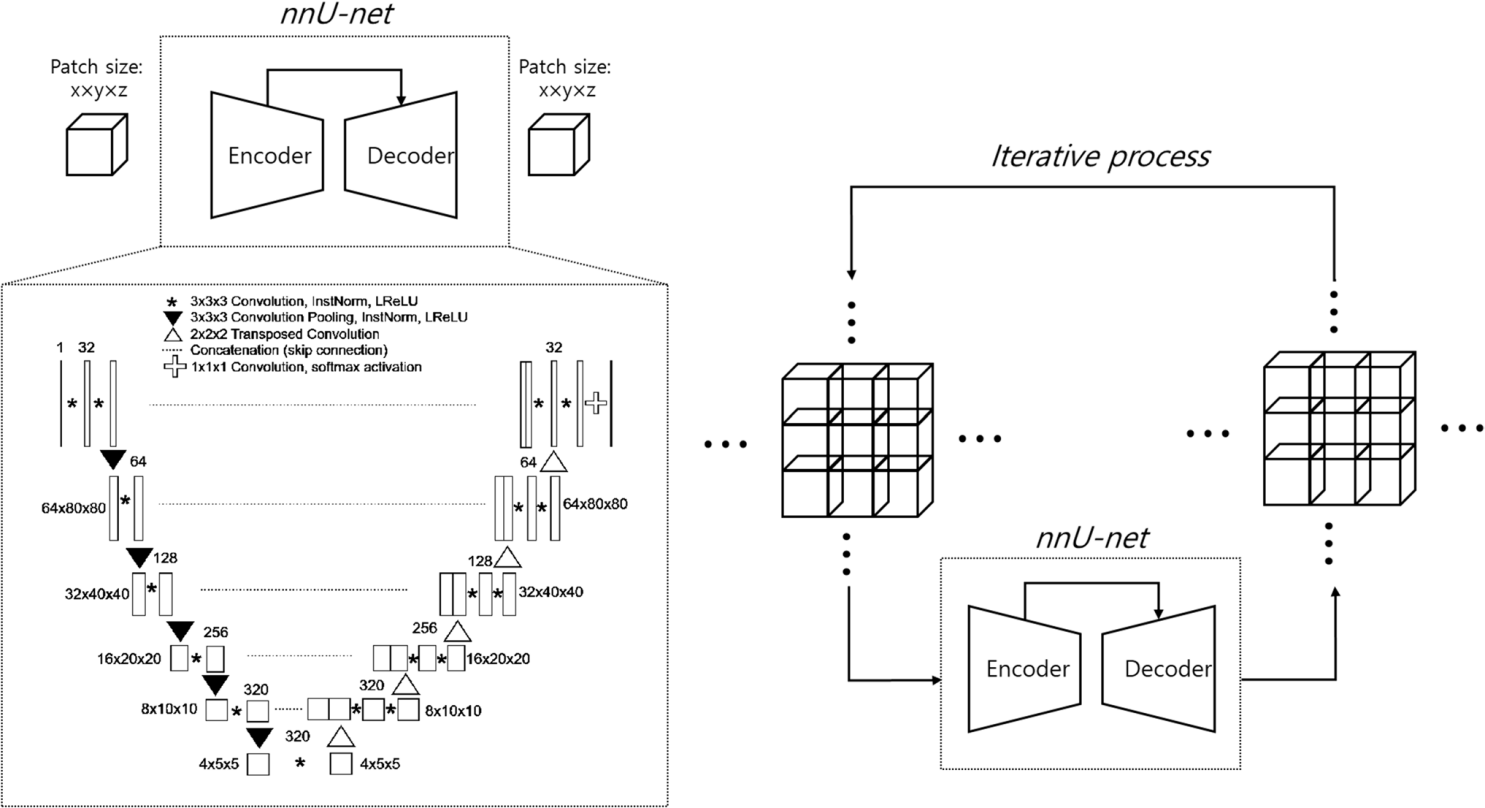
Architecture of our 3D nnU-Net-based multi-organ segmentation model. InstNorm = instance normalization, LReLU = leaky rectified linear unit

Two separate 3D nnU-Net models were developed for multi-organ segmentation in the abdominal CT and low-dose chest CT scans (Fig. 1). The development datasets of each model were randomly categorized into training, tuning, and internal test sets with 85, 10, and 10 scans, respectively, for abdominal CT, and 40, 10, and 10 scans, respectively, for low-dose chest CT. Each model was designed to consider internal organ areas, predicted using a body composition segmentation algorithm [24] as input. Subsequently, the model output segmented areas of the liver, spleen, right kidney, and left kidney in abdominal CT and the liver and spleen in low-dose chest CT. The 3D nnU-Net network determined the preprocessing methods and network hyperparameters based on the dataset (including patch size, number of pooling layers, and convolutional kernel size). The patch size was initialized to the median image shape and iteratively reduced to adapt the network topology until the network could be trained with a batch size of at least two, considering the memory constraints of the graphics processing unit. The final patch size configurations were 56 × 192 × 160 and 96 × 160 × 160 for the abdominal CT and low-dose chest CT models, respectively. The last activation function was softmax, and the loss function was the sum of the Dice and cross-entropy losses. Stochastic gradient descent (Nesterov momentum = 0.99) was the optimization algorithm. The polynomial learning rate scheduler was initialized at 0.01. The models were trained for 1000 epochs.

### Organ volumetry and 3D radiomics feature extraction

The clinical applicability of the developed models was evaluated by testing their accuracy in measuring organ volume and extracting radiomics features of the target organ. Organ volume and volume-based radiomics features were assessed for each target organ. These radiomics features included three shape-based parameters (sphericity, elongation, and flatness) and seven first-order statistics (mean Hounsfield unit [HU], median HU, standard deviation, skewness, kurtosis, uniformity, and entropy). These features were extracted from the automatically segmented masks generated by the developed models and the GT masks.

### Statistical analysis

Three widely used metrics for evaluating medical imaging segmentation—including Dice similarity coefficients (DSCs), sensitivity, and precision—were calculated to evaluate the accuracy of the automated segmentation performed by the developed model. These metrics were separately computed into the internal and external test sets by comparing the model-derived masks with the corresponding GT masks [25].

In the external test set, the agreements in organ volumes and radiomics features between the model-derived and the GT masks were evaluated using the intraclass correlation coefficient (ICC) and Bland–Altman analysis. ICC values below 0.5, 0.5–0.75, 0.75–0.9, and above 0.9 were categorized as poor, moderate, good, and excellent agreements, respectively [26]. Furthermore, the volume differences between the model-derived and GT masks were examined. A volume difference below 5% was considered an accurate estimation. Conversely, differences exceeding 5% were categorized as an overestimation or underestimation. Additionally, we determined whether there were significant differences in organ volume and radiomic features measured from model-derived and GT masks using paired t-tests. The significance level was set at p < 0.05.

All statistical analyses were performed using the R statistical software (version 4.2.0; R Foundation for Statistical Computing, Vienna, Austria).

## Results

### Segmentation performance

The fully-automated 3D nnU-Net models developed for abdominal CT and low-dose chest CT scans in this study demonstrated excellent performance in the multi-organ segmentation of abdominal solid organs. The mean DSC value for each assessed organ exceeded 0.95 in the internal and external test sets (Table 1). In the internal test set, the mean DSCs were 0.982, 0.973, 0.966, and 0.967 for the liver, spleen, right kidney and left kidney, respectively, in the abdominal CT scans, and 0.969 and 0.962 for the liver and spleen, respectively, in the low-dose chest CT scans. In the external test set, the mean DSCs were 0.968, 0.960, 0.952, and 0.958 for the liver, spleen, right kidney and left kidney, respectively, in the abdominal CT scans, and 0.969 and 0.960 for the liver and spleen, respectively, in the low-dose chest CT scans. The mean sensitivity and precision values of each organ in the internal test set varied across the target organs, ranging between 0.969–0.983 and 0.963–0.981, respectively, in abdominal CT scans, and between 0.962–0.980 and 0.959–0.962, respectively, in low-dose chest CT scans. In the external test set, the mean sensitivity and precision values of each organ ranged between 0.959–0.974 and 0.946–0.963, respectively, in abdominal CT scans and 0.957–0.980 and 0.959–0.963, respectively, in low-dose chest CT scans. Table 1 presents detailed results regarding the segmentation accuracy of the developed models.

**Table 1:** Segmentation performance of our 3D nnU-net-based models

	Abdominal CT model				Low-dose chest CT model	
	Liver	Spleen	Right kidney	Left kidney	Liver	Spleen
Internal test set						
DSC	0.982 ± 0.002	0.973 ± 0.007	0.966 ± 0.006	0.967 ± 0.007	0.969 ± 0.008	0.962 ± 0.006
Sensitivity	0.983 ± 0.007	0.969 ± 0.010	0.970 ± 0.007	0.971 ± 0.006	0.980 ± 0.005	0.962 ± 0.012
Precision	0.981 ± 0.005	0.978 ± 0.009	0.963 ± 0.015	0.964 ± 0.013	0.959 ± 0.015	0.962 ± 0.018
External test set						
DSC	0.968 ± 0.012	0.960 ± 0.018	0.952 ± 0.014	0.958 ± 0.009	0.969 ± 0.006	0.960 ± 0.012
Sensitivity	0.974 ± 0.019	0.961 ± 0.024	0.959 ± 0.016	0.965 ± 0.012	0.980 ± 0.008	0.957 ± 0.021
Precision	0.963 ± 0.014	0.959 ± 0.020	0.946 ± 0.023	0.951 ± 0.017	0.959 ± 0.014	0.963 ± 0.008

Data are presented as mean ± standard deviation

*DSC* Dice similarity coefficient

### Organ volumetry

**Fig. 2:**
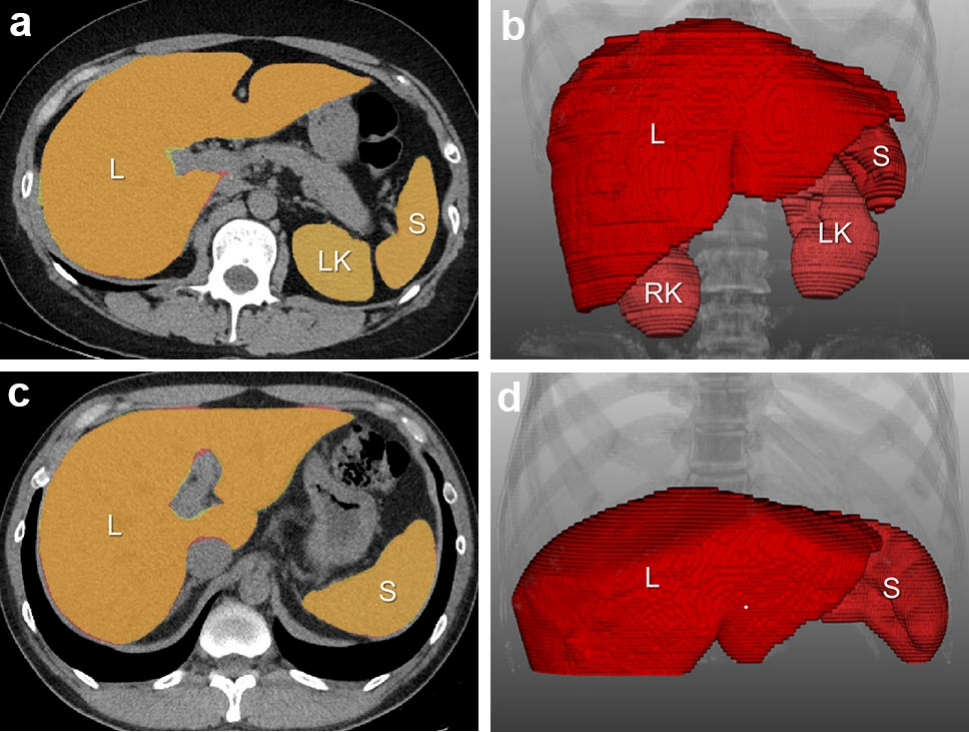
Illustrations of abdominal organ segmentation in **a**, **b** non-enhanced abdominal CT images and **c**, **d** low-dose chest CT images. **a**, **c** In the representative 2D axial images, the model-derived mask (red) closely matches the ground-truth mask (yellow) for each target organ, so most of the organs appears as orange color due to the overlap between the two masks. **b**, **d** The 3D reconstruction images of the model-derived masks (red) demonstrate good segmentation accuracy. L = liver, S = spleen, RK = right kidney, LK = left kidney

The developed segmentation model achieved accurate organ volume estimations (< 5% difference from the GT) in 98.2% (54/55), 90.9% (50/55), 83.6% (46/55), and 94.5% (52/55) of cases for the liver, spleen, right kidney, and left kidney in the abdominal CT scans, respectively, and 90.0% (9/10) for the liver and spleen in the low-dose chest CT scans (Supplementary Table 3). Figure 2 illustrates examples of organ volume estimations performed using the developed models.

The model-estimated organ volumes showed small mean differences from the GT measurements of 1.2%, 0.2%, 1.4%, and 1.5% for the liver, spleen, right kidney, and left kidney, respectively, in abdominal CT scans, and 2.2% and -0.7%, for the liver and spleen, respectively, in low-dose chest CT scans (Supplementary Fig. 1). While these differences were found to be significant for the liver, right kidney, and left kidney in abdominal CT (p < 0.001, 0.004, and < 0.001, respectively) and for the liver in low-dose chest CT (p = 0.006), there was no statistically significant difference observed for the spleen in either abdominal or low-dose chest CT scans (p = 0.674 and 0.401, respectively). Additionally, the ICCs between the model estimations and GT indicated excellent agreements for each target organ as follows: 0.994, 0.998, 0.986, and 0.986 for the liver, spleen, right kidney, and left kidney, respectively, in the abdominal CT scans, and 0.992 and 0.997 for the liver and spleen, respectively, in the low-dose chest CT scans (Table 2).

**Table 2:** Agreement between model-derived measurements and ground-truth measurements in external test set

ICC (95% CI)	Abdominal CT model				Low-dose chest CT model	
	Liver	Spleen	Right kidney	Left kidney	Liver	Spleen
Organ volume	0.994 (0.984–0.997)	0.998 (0.997–0.999)	0.986 (0.972–0.990)	0.986 (0.959–0.994)	0.992 (0.878–0.999)	0.997 (0.989–0.999)
Volumetric radiomics features						
Mean HU	0.999 (0.996–1.000)	0.976 (0.945–0.988)	0.983 (0.971–0.990)	0.970 (0.849–0.989)	0.995 (0.981–0.999)	0.979 (0.567–0.996)
Median HU	0.999 (0.999–1.000)	0.997 (0.994–0.998)	0.996 (0.994–0.998)	0.994 (0.989–0.997)	0.998 (0.993–1.000)	0.996 (0.972–0.999)
Standard deviation	0.860 (0.438–0.946)	0.728 (0.374–0.868)	0.883 (0.799–0.932)	0.785 (0.218–0.919)	0.877 (0.441–0.971)	0.793 (-0.061–0.959)
Skewness	0.328 (-0.039–0.597)	0.831 (0.572–0.920)	0.794 (0.595–0.890)	0.424 (0.187–0.616)	0.454 (-0.102–0.821)	0.210 (-0.083–0.645)
Kurtosis	0.196 (-0.047–0.427)	0.933 (0.887–0.961)	0.683 (0.425–0.823)	0.053 (-0.203–0.307)	0.373 (-0.137–0.777)	0.152 (-0.086–0.563)
Uniformity	0.998 (0.996–0.999)	0.993 (0.988–0.996)	0.992 (0.987–0.995)	0.990 (0.975–0.995)	0.993 (0.970–0.998)	0.985 (0.480–0.997)
Entropy	0.993 (0.975–0.997)	0.977 (0.952–0.988)	0.982 (0.969–0.989)	0.968 (0.857–0.988)	0.966 (0.822–0.992)	0.931 (0.041–0.988)
Sphericity	0.445 (0.148–0.655)	0.819 (0.513–0.918)	0.399 (0.058–0.636)	0.661 (0.308–0.825)	0.368 (-0.066–0.796)	0.427 (-0.090–0.828)
Elongation	0.992 (0.986–0.995)	0.983 (0.968–0.990)	0.992 (0.985–0.995)	0.992 (0.987–0.996)	0.991 (0.884–0.998)	0.978 (0.914–0.995)
Flatness	0.990 (0.983–0.995)	0.973 (0.954–0.984)	0.992 (0.986–0.995)	0.994 (0.989–0.996)	0.997 (0.985–0.999)	0.987 (0.948–0.997)

*ICC* intraclass correlation coefficient; *CI* confidence interval; *HU* Hounsfield unit

### Radiomics features

The volumetric mean HU—a commonly used radiomics feature for organ characterization—of the model-derived masks demonstrated very small mean differences below 1.0 HU from the GT for all target organs in abdominal CT scans. In low-dose chest CT scans, the mean differences were 0.3 and 1.2 HU for the liver and spleen, respectively (Supplementary Fig. 2). Detailed comparisons between the model and GT-derived measurements are presented in Supplementary Table 4. Moreover, the ICCs for the mean HU of the segmented organs by the developed model and the GT exceeded 0.95 for all target organs in abdominal and low-dose chest CT scans (Table 2).

The model-estimated measurements of median HU, uniformity, entropy, elongation, and flatness demonstrated excellent agreement (all ICCs > 0.9) with the GT measurements for all target organs in abdominal and low-dose chest CT scans (Table 2). Standard deviation showed moderate-to-good agreement (ICC, 0.728–0.883) in abdominal CT scans and good agreement (ICC, 0.793–0.877) in low-dose chest CT scans.

**Fig. 3:**
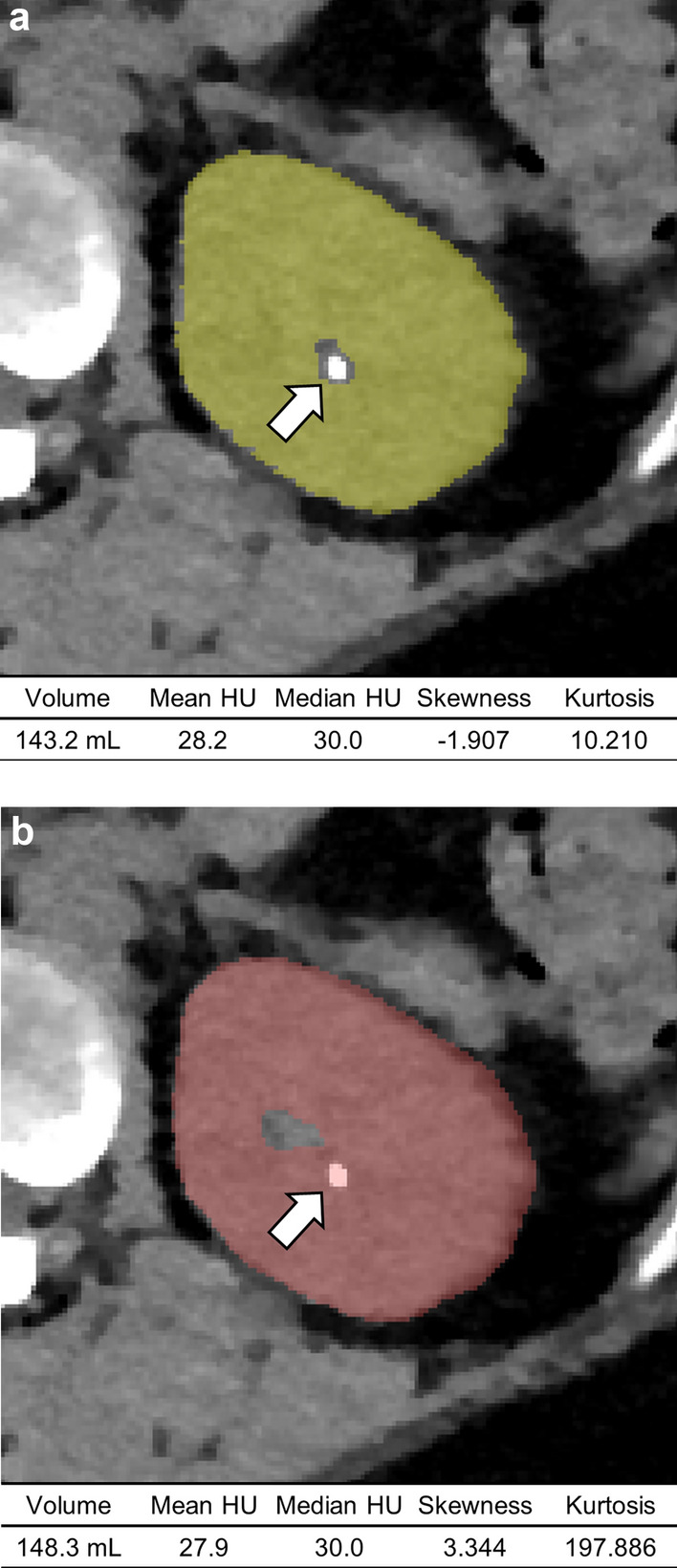
An example case demonstrating substantial differences in certain radiomics features between **a** ground-truth and **b** model-derived masks. The ground-truth mask for the left kidney (in yellow, a) closely matches the model-derived mask (in red, b) but excludes a small calyceal stone (arrow). Notably, volume and mean Hounsfield unit (HU), as well as median HU, show negligible differences between the two methods. However, significant discrepancies are seen in skewness (− 1.907 versus − 3.344) and kurtosis values (10.210 versus 197.886), possibly attributed to the presence or absence of the high attenuation stone within the mask.

In contrast, agreements in skewness (ICC, 0.328–0.831 in abdominal CT and 0.210–0.454 in low-dose chest CT scans), kurtosis (ICC, 0.053–0.933 in abdominal CT and 0.152–0.373 in low-dose chest CT scans), and sphericity (ICC, 0.399–0.819 in abdominal CT and 0.368–0.427 in low-dose chest CT scans) between model-estimated measurements and GTs were inconsistent depending on the target organs (Fig. 3). Some of these measurements had great differences with wide ranges of 95% limit of agreements (Supplementary Table 4).

## Discussion

Our multi-organ segmentation models, developed based on the 3D nnU-Net architecture, demonstrated excellent performance in segmenting abdominal solid organs in non-enhanced abdominal CT and low-dose chest CT scans. The mean DSCs for our models exceeded 0.95 in the external test set for all target organs, including the liver, spleen, right kidney, and left kidney in abdominal CT and the liver and spleen in low-dose chest CT. Furthermore, we assessed the usefulness of our models for estimating organ volumes and extracting 3D radiomics features from CT scans. Our models accurately measured the volume of each organ on the abdominal and low-dose chest CT images owing to their precise segmentation capability, exhibiting an error below 5% from the GT volumes in most cases. Our models reliably extracted certain shape features and first-order statistics from the segmented organs, displaying excellent agreement with the GT measurements.

In our multi-organ segmentation models, we used the Leaky ReLU activation function, a combination of Dice and cross-entropy losses, and the Stochastic Gradient Descent algorithm for the activation function, loss function, and optimization algorithm, respectively. These choices were based on experimental results from the nnU-Net paper [20]. Especially, they facilitate fast initial convergence and fine-tuning during training, while also improving class imbalance handling, training stability, and segmentation accuracy.

Despite the challenges posed by non-enhanced CT images in delineating anatomical boundaries, the segmentation performance of our model using 3D nnU-Net was comparable with that of recently reported convolutional neural network-based models designed for contrast-enhanced CT scans. These models achieved mean DSCs of 0.94–0.97 for the liver, spleen, or kidneys [27–29]. Notably, our model’s performance is similar to a prior model specifically fine-tuned for non-enhanced CT, which was developed by training on a large volume of contrast-enhanced CT scans and then adapted using a small number of non-contrast CT images, achieving DSCs of 0.96 ± 0.01 for the liver and 0.94 ± 0.03 for the spleen [30]. The nnU-net has been recognized for its robust organ segmentation capability, as evidenced by its success in a recent kidney segmentation challenge involving contrast-enhanced CT scans [29]. Our results further highlighted the efficacy of this architecture in non-enhanced CT scans. Additionally, our utilization of internal organ masks obtained through a body composition segmentation algorithm rather than employing entire CT images as inputs may have contributed to developing our high-performance models.

Recent studies on automated segmentation models for medical imaging have revealed remarkable accuracy, primarily focusing on the liver and spleen volume estimation. For example, in a study involving contrast-enhanced CT, a model demonstrated 95% limit of agreement of 0.2 ± 3.1% for the liver and − 0.6 ± 3.8% for the spleen compared with the GT volumes [28]. Similarly, a model applied to hepatobiliary MRI reported 0.1 ± 3.7% and 0.2 ± 7.9% for the liver and spleen, respectively [31]. Our model for non-enhanced abdominal CT exhibited a performance comparable with those for the liver and spleen volume estimation in these studies while achieving high accuracy in measuring kidney volumes. Furthermore, our model accurately measured the liver and spleen volumes using low-dose chest CT. This method does not capture the complete organ volumes because of the limited coverage of most low-dose chest CT scans. Consequently, utilizing this as a direct marker of organ volume might be inappropriate. Nonetheless, our model can serve as an indicator, providing insights into the degree to which organ volume contributes to radiomics analyses using low-dose chest CT.

One of the most widely utilized radiomics features obtained from non-enhanced abdominal CT images is the assessment of fatty liver based on the mean HU value of the liver. Currently, the volumetric mean HU of the liver obtained through automated segmentation is actively employed in population-based studies for detecting and assessing the severity of fatty liver [32, 33]. Our study supports the reliability of this automated approach by demonstrating the excellent agreements in the mean HU values of the liver between the model-derived and GT masks. Moreover, our study demonstrated that automated segmentation on low-dose chest CT can reliably measure the mean HU of the liver within the scan range, which provides fundamental evidence for evaluating fatty livers using low-dose chest CT in the future. Considering the growing utilization of low-dose chest CT for lung cancer screening and medical check-ups, low-dose chest CT holds promise for assessing fatty liver in such contexts. This may mitigate the need for additional abdominal CT examinations in patients who have undergone low-dose chest CT for these purposes.

In addition to mean HU, several 3D radiomics features, including median HU, uniformity, entropy, elongation, and flatness, showed strong agreement between the model-derived and GT masks. However, certain features, such as skewness, kurtosis, and sphericity, displayed inconsistencies, as indicated by their lower ICCs and wider 95% limit of agreements. Given the high segmentation accuracy achieved by our model, these features, which exhibit reduced reliability, appear to be sensitive to minor outliers. Skewness and kurtosis are inherently sensitive to even moderate outliers in a histogram [34]. Even a small number of voxels with extremely high or low HU values (e.g., calcifications or air) can significantly affect these metrics, likely resulting in lower ICC values. Sphericity, calculated as the ratio of surface area to volume, can also be impacted by minor discrepancies between the ground-truth and model-derived masks, particularly at the surface of the organ. These surface-level differences, though small, can significantly influence the sphericity value. Additionally, the increased image noise in low-dose CT may further amplify the effects of these small discrepancies. Therefore, systematically addressing errors stemming from outliers and ensuring the robustness of the radiomics analysis measurements is crucial to effectively utilizing these radiomics features. Nevertheless, evaluating whether removing such outliers will affect the faithful representation of the actual organ characteristics is necessary. Alternatively, adopting the principles of the Image Biomarker Standardization Initiative [35], which discriminates reliably obtainable radiomics features, could help identify substantively applicable biomarker candidates. Our results, which revealed feature-dependent reliability, support the necessity of such discrimination in radiomics derived from organ segmentation.

Our study had some limitations. First, the number of CT scans in the external test set was relatively small. Larger volumes of data from various CT scanners are necessary for further validation. Second, the CT scans used for developing and validating primarily featured normal or near-normal abdominal organs. Diseased organs may exhibit distinct shapes or CT attenuation compared with normal organs. Therefore, additional training and validation involving cases of diseased organs may be essential to effectively utilize our model in diverse clinical settings. Third, the scan range of the low-dose chest CT for the upper abdomen is typically incomplete, and portions of the liver and spleen may not be included. However, since the comparison between the ground truth and model-derived masks was performed using the same CT images, the evaluation remains fair between the two methods. Finally, the observed variation in reliability, which depends on the types of radiomics features obtained from automated segmentation in our study, may require testing across multiple segmentation algorithms. This extended investigation is critical for establishing the generalizability of these findings and providing guidance for radiomics feature selection in studies using automated segmentation.

In conclusion, our 3D nnU-Net based models accurately segmented abdominal solid organs in both non-enhanced abdominal CT and low-dose chest CT scans. The automated segmentation provided organ volumes and various radiomics features with high accuracy, closely matching those obtained from manual segmentation. This indicates that our model can be effectively utilized in clinical and research settings that require organ volumetry or radiomics, significantly reducing the time required for manual segmentation. While some radiomics features were found to be unreliable, our findings can guide the selection of appropriate features in radiomics studies using automated segmentation. Future work should focus on improving the reliability of these features and exploring the model's applicability to other organ systems.

## Supplementary Information

Below is the link to the electronic supplementary material.

Supplementary file1 (DOCX 423 KB)

## Data Availability

The artificial intelligence algorithm developed from this study is available through the commercial product (MEDIP PRO, MEDICALIP Co. Ltd., Seoul, Korea) and the code can be available from the corresponding author on reasonable request.
