# Supplementary material for: Automated abdominal organ segmentation algorithms for non-enhanced CT for volumetry and 3D radiomics analysis
Source: Abdom Radiol (NY). 2024 Sep 19;50(3):1448–56. doi: 10.1007/s00261-024-04581-5 (PMC11821665; doi:10.1007/s00261-024-04581-5)
Supplement: Supplementary file 1 — Supplementary file1 (DOCX 423 KB) [file 261_2024_4581_MOESM1_ESM.docx]

**Supplementary table 1. CT acquisition and reconstruction parameters of non-enhanced abdominal CT**

|  | Development set | External test set | | | | |
| --- | --- | --- | --- | --- | --- | --- |
| Machine | Aquilion ONE  (Canon) | SOMATOM Force  (Siemens) | IQon Spectral CT  (Philips) | iCT 256  (Philips) | Revolution CT  (GE) | SOMATOM Definition  (Siemens) |
| Number of subjects | 105 | 15 | 12 | 10 | 9 | 9 |
| Collimation width (mm) | 0.5 × 80-100 | 0.6 × 64-96 | 0.625 × 64 | 0.625 × 64 | 0.625 × 128 | 1.2 × 24 or 0.6 × 32 |
| Tube voltage (kVp) | 80-120 | 80-100 | 100-120 | 120 | 80 | 100 |
| Tube current (mAs) | ATCM | ATCM | ATCM | ATCM | ATCM | ATCM |
| Slice thickness (mm) | 3.0 | 3.0 | 3.0 | 3.0 | 2.5 | 3.0 |
| Slice interval (mm) | 3.0 | 2.0 | 2.0 | 3.0 | 2.5 | 2.0 |
| Reconstruction method | AIDR | ADMIRE | IMR | iDose | TrueFidelity | FBP |
| Kernel | Soft | Soft | Soft | Soft | Soft | Soft |

ADMIRE, advanced modeled iterative reconstruction; AIDR, adaptive iterative dose reduction; ATCM, automatic tube current modulation; FBP, filtered back projection; IMR, iterative model reconstruction

**Supplementary table 2. CT acquisition and reconstruction parameters of low-dose chest CT**

|  | Development set | | External test set | |
| --- | --- | --- | --- | --- |
| Machine | SOMATOM Force  (Siemens) | SOMATOM Definition  (Siemens) | iCT 256  (Philips) | Discovery CT750 HD  (GE) |
| Number of subjects | 25 | 20 | 15 | 10 |
| Collimation width (mm) | 0.6 × 96 | 0.6 × 32 | 0.625 × 64 | 0.625 × 64 |
| Tube voltage (kVp) | 100 | 120 | 120 | 120 |
| Tube current (mAs) | 150^†^ or ATCM | 30 | 20 | 38-58 |
| Slice thickness (mm) | 3.0 | 3.0 | 3.0 | 2.5 |
| Slice interval (mm) | 3.0 | 3.0 | 3.0 | 2.5 |
| Reconstruction method | ADMIRE | FBP | iDose | ASIR |
| Kernel | Soft | Soft | Soft | Soft |

^†^Achieving dose reduction by increasing pitch (3.00) rather than reducing tube current.

ADMIRE, advanced modeled iterative reconstruction; ASIR, adaptive statistical iterative reconstruction; ATCM, automatic tube current modulation; FBP, filtered back projection

**Supplementary table 3. Performance of the developed algorithm for organ volume estimation**

|  | Accurate estimation^*^ | Under-estimation | Over-estimation |
| --- | --- | --- | --- |
| Abdominal CT |  |  |  |
| Liver | 98.2% (54/55) | 0% (0/55) | 1.8% (1/55) |
| Spleen | 90.9% (50/55) | 7.3% (4/55) | 1.8% (1/55) |
| Right kidney | 83.6% (46/55) | 14.5% (8/55) | 1.8% (1/55) |
| Left kidney | 94.5% (52/55) | 5.5% (3/55) | 0% (0/55) |
| Low-dose chest CT |  |  |  |
| Liver | 90.0% (9/10) | 10.0% (1/10) | 0% (0/10) |
| Spleen | 90.0% (9/10) | 0% (0/10) | 10.0% (1/10) |

Note. Data in parentheses are numbers calculate the percentages.

^*^Accurate estimation was within ± 5% of the ground-truth volume, while over-estimation and under-estimation exceeded +5% and -5%, respectively.

**Supplementary table 4. Volumetric radiomics features of model-derived and ground-truth measurements**

|  | Abdominal CT | | | Low-dose chest CT | | |
| --- | --- | --- | --- | --- | --- | --- |
|  | Model-derived | Ground truth | 95% LOA | Model-derived | Ground truth | 95% LOA |
| Liver | | | | | | |
| Mean HU | 53.9 ± 10.5 | 54.2 ± 10.5 | -0.3 ± 0.8 | 53.1 ± 7.4 | 52.8 ± 7.6 | 0.3 ± 1.4 |
| Median HU | 54.9 ± 10.7 | 55.0 ± 10.7 | -0.1 ± 0.7 | 53.7 ± 7.5 | 53.7 ± 7.5 | 0.0 ± 0.9 |
| Standard deviation (HU) | 16.3 ± 3.3 | 15.1 ± 3.3 | 1.2 ± 2.6 | 20.3 ± 2.5 | 21.2 ± 2.9 | -0.9 ± 2.2 |
| Skewness | -2.333 ± 1.724 | -1.059 ± 0.705 | -1.273 ± 2.636 | -0.291 ± 0.186 | -0.571 ± 0.448 | 0.280 ± 0.612 |
| Kurtosis | 33.566 ± 31.562 | 11.453 ± 12.515 | 22.112 ± 56.594 | 3.918 ± 0.697 | 5.208 ± 2.084 | -1.290 ± 3.097 |
| Uniformity | 0.110 ± 0.028 | 0.110 ± 0.028 | -0.001 ± 0.003 | 0.073 ± 0.010 | 0.072 ± 0.010 | 0.001 ± 0.002 |
| Entropy | 3.540 ± 0.354 | 3.516 ± 0.357 | 0.024 ± 0.069 | 4.050 ± 0.187 | 4.081 ± 0.020 | -0.031 ± 0.084 |
| Sphericity | 0.542 ± 0.025 | 0.525 ± 0.031 | 0.016 ± 0.054 | 0.605 ± 0.043 | 0.548 ± 0.030 | 0.058 ± 0.045 |
| Elongation | 0.597 ± 0.066 | 0.598 ± 0.064 | -0.002 ± 0.016 | 0.608 ± 0.064 | 0.602 ± 0.061 | 0.006 ± 0.012 |
| Flatness | 0.459 ± 0.049 | 0.460 ± 0.049 | -0.001 ± .013 | 0.435 ± 0.068 | 0.432 ± 0.067 | 0.003 ± 0.009 |
| Spleen | | | | | | |
| Mean HU | 47.2 ± 5.8 | 47.8 ± 5.2 | -0.6 ± 2.1 | 41.8 ± 7.0 | 40.6 ± 7.0 | 1.2 ± 1.7 |
| Median HU | 49.8 ± 5.5 | 49.9 ± 5.3 | -0.1 ± 0.8 | 42.7 ± 6.9 | 42.3 ± 6.9 | 0.4 ± 1.0 |
| Standard deviation (HU) | 20.5 ± 4.6 | 18.6 ± 3.9 | 1.9 ± 5.3 | 22.0 ± 3.2 | 24.1 ± 3.8 | -2.1 ± 2.5 |
| Skewness | -2.182 ± 2.595 | -1.307 ± 2.636 | -0.875 ± 2.559 | -0.328 ± 0.142 | -0.784 ± 0.305 | 0.456 ± 0.425 |
| Kurtosis | 26.252 ± 59.378 | 19.933 ± 75.649 | 6.319 ± 47.609 | 3.606 ± 0.413 | 5.403 ± 1.174 | -1.797 ± 1.789 |
| Uniformity | 0.102 ± 0.027 | 0.103 ± 0.028 | -0.001 ± 0.006 | 0.068 ± 0.011 | 0.066 ± 0.012 | 0.002 ± 0.002 |
| Entropy | 3.725 ± 0.368 | 3.692 ± 0.361 | 0.033 ± 0.139 | 4.158 ± 0.225 | 4.237 ± 0.241 | -0.079 ± 0.083 |
| Sphericity | 0.611 ± 0.034 | 0.597 ± 0.041 | 0.014 ± 0.037 | 0.649 ± 0.022 | 0.611 ± 0.034 | 0.038 ± 0.037 |
| Elongation | 0.707 ± 0.080 | 0.702 ± 0.079 | 0.005 ± 0.028 | 0.677 ± 0.062 | 0.677 ± 0.062 | 0.000 ± 0.027 |
| Flatness | 0.417 ± 0.051 | 0.416 ± 0.052 | 0.001 ± 0.024 | 0.435 ± 0.065 | 0.435 ± 0.063 | 0.000 ± 0.022 |
| Right kidney | | | | | | |
| Mean HU | 30.8 ± 4.7 | 30.9 ± 4.6 | -0.1 ± 1.7 | N/A | | |
| Median HU | 33.4 ± 4.7 | 33.4 ± 4.4 | -0.0 ± 0.8 |  |  |  |
| Standard deviation (HU) | 20.0 ± 3.9 | 19.5 ± 3.6 | 0.6 ± 3.4 |  |  |  |
| Skewness | -1.687 ± 0.762 | -1.466 ± 0.667 | -0.221 ± 0.819 |  |  |  |
| Kurtosis | 9.447 ± 4.129 | 7.984 ± 3.138 | 1.463 ± 5.241 |  |  |  |
| Uniformity | 0.095 ± 0.024 | 0.095 ± 0.024 | 0.000 ± 0.006 |  |  |  |
| Entropy | 3.798 ± 0.337 | 3.791 ± 0.327 | 0.007 ± 0.125 |  |  |  |
| Sphericity | 0.560 ± 0.028 | 0.536 ± 0.039 | 0.025 ± 0.066 |  |  |  |
| Elongation | 0.566 ± 0.048 | 0.568 ± 0.049 | -0.002 ± 0.011 |  |  |  |
| Flatness | 0.403 ± 0.045 | 0.404 ± 0.046 | -0.001 ± 0.011 |  |  |  |
| Left kidney | | | | | | |
| Mean HU | 30.4 ± 4.9 | 31.2 ± 4.6 | -0.8 ± 1.8 | N/A | | |
| Median HU | 33.0 ± 4.9 | 33.2 ± 4.8 | -0.2 ± 1.0 |  |  |  |
| Standard deviation (HU) | 20.8 ± 3.7 | 18.5 ± 3.3 | 1.7 ± 3.4 |  |  |  |
| Skewness | -1.537 ± 0.980 | -1.287 ± 0.578 | -0.250 ± 1.669 |  |  |  |
| Kurtosis | 12.722 ± 25.906 | 7.118 ± 2.748 | 5.604 ± 49.644 |  |  |  |
| Uniformity | 0.095 ± 0.023 | 0.096 ± 0.023 | -0.002 ± 0.006 |  |  |  |
| Entropy | 3.811 ± 0.322 | 3.759 ± 0.319 | 0.052 ± 0.125 |  |  |  |
| Sphericity | 0.557 ± 0.031 | 0.540 ± 0.036 | 0.017 ± 0.048 |  |  |  |
| Elongation | 0.548 ± 0.053 | 0.548 ± 0.052 | 0.000 ± 0.013 |  |  |  |
| Flatness | 0.441 ± 0.050 | 0.441 ± 0.049 | 0.001 ± 0.011 |  |  |  |

HU, Hounsfield unit; LOA, limits of agreement; N/A, not applicable

**Supplementary figure 1. Bland-Altman plots comparing model-estimated and ground-truth organ volumes in non-enhanced abdominal CT (a) and low-dose chest CT (b).** Black line = mean difference, red dotted line = 95% limits of agreement. ABCT = abdominal CT, LDCT = low-dose chest CT, GT = ground truth

**
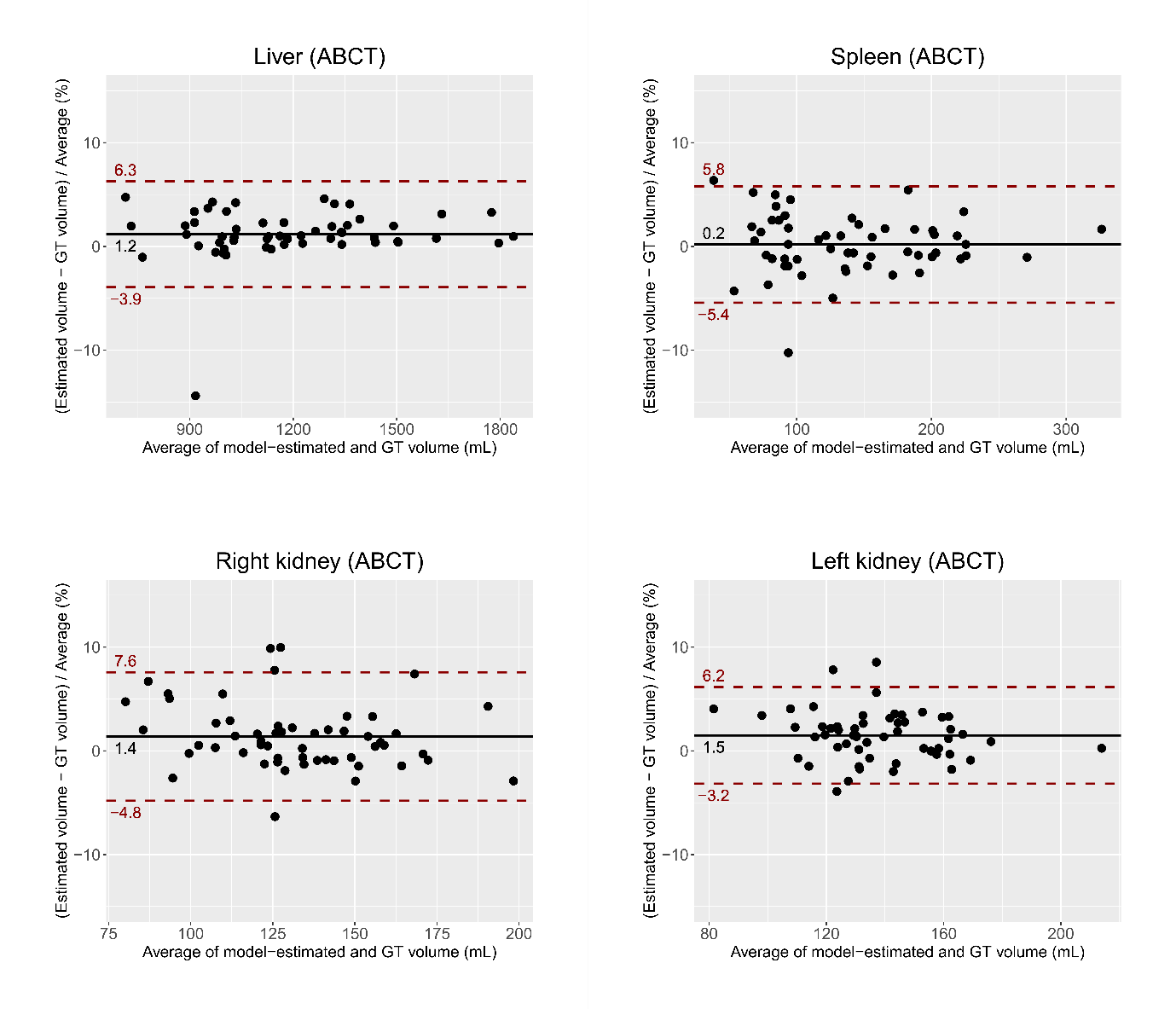
**

**(a)**

**
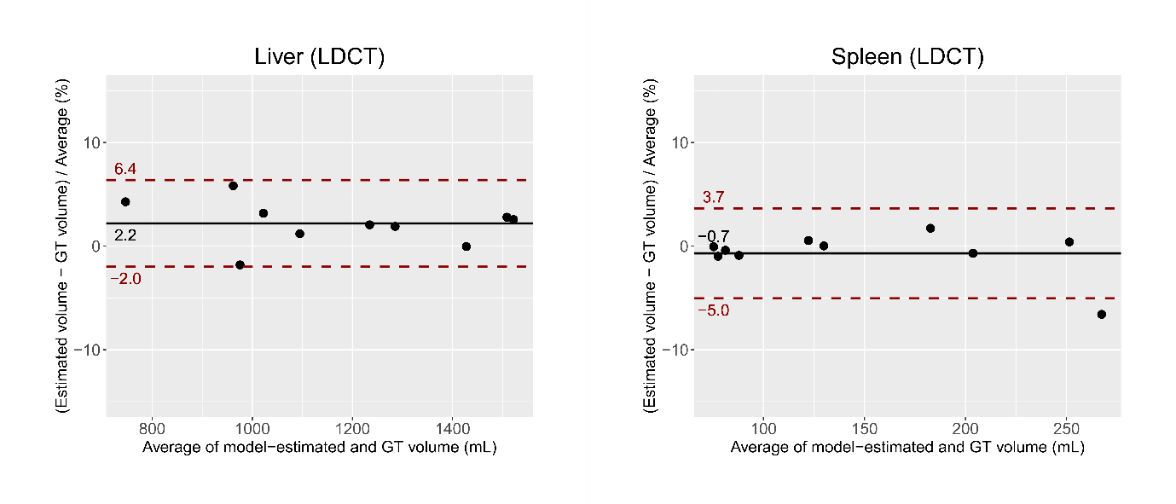
**

**(b)**

**Supplementary figure 2. Bland-Altman plots comparing volumetric mean Hounsfield units measured by model-derived and ground-truth masks in non-enhanced abdominal CT (a) and low-dose chest CT (b).** Black line = mean difference, red dotted line = 95% limits of agreement. ABCT = abdominal CT, LDCT = low-dose chest CT, GT = ground truth, HU = Hounsfield unit

**
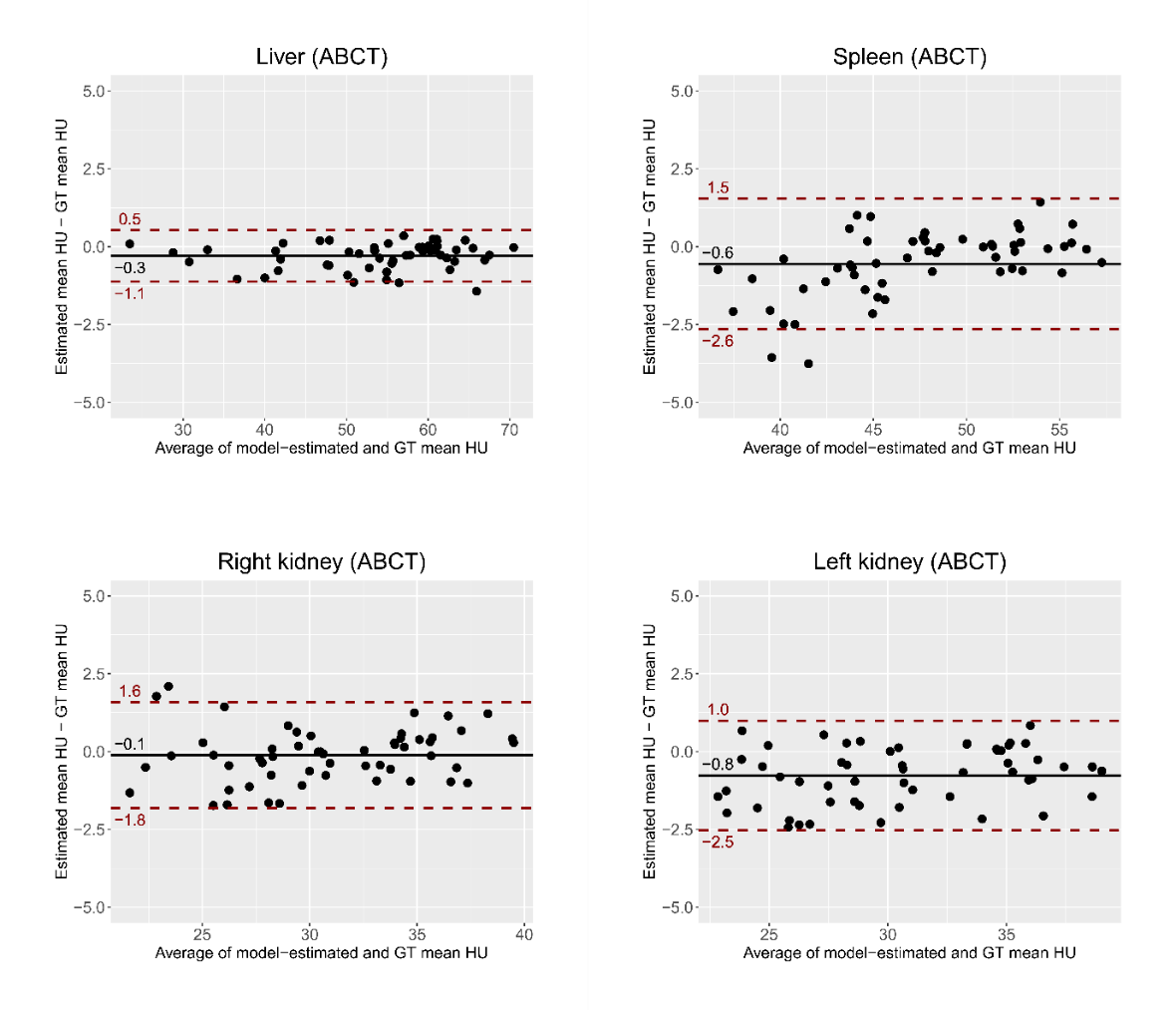
**

**(a)**

**
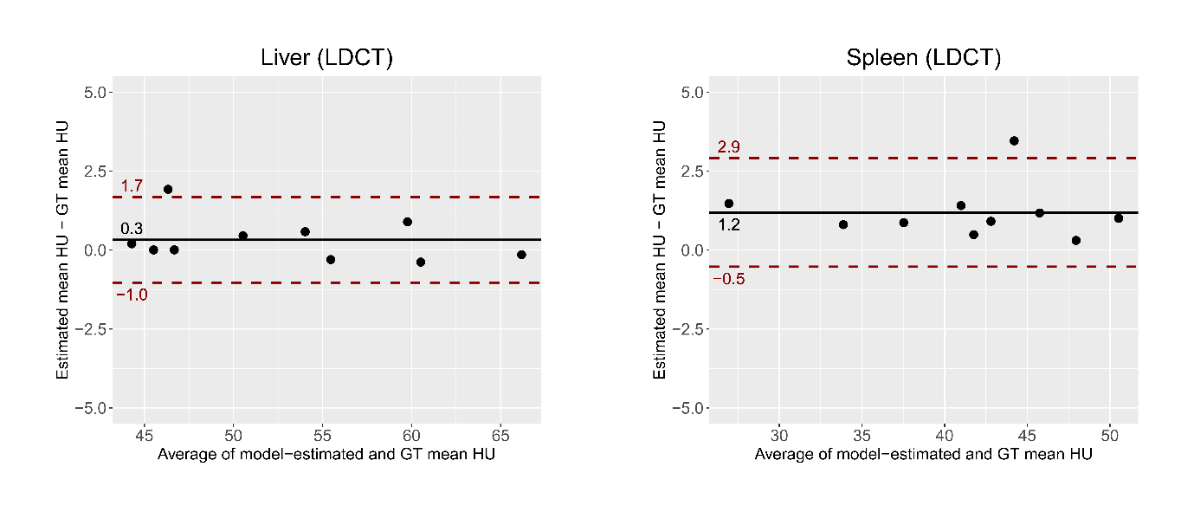
**

**(b)**
